# The Mathematical Model of the Bcl-2 Family Mediated MOMP Regulation Can Perform a Non-Trivial Pattern Recognition

**DOI:** 10.1371/journal.pone.0081861

**Published:** 2013-12-26

**Authors:** Tomas Tokar, Jozef Ulicny

**Affiliations:** Department of Biophysics, University of P. J. Safarik in Kosice, Kosice, Slovakia; University Freiburg, Germany

## Abstract

Interactions between individual members of the B-cell lymphoma 2 (Bcl-2) family of proteins form a regulatory network governing mitochondrial outer membrane permeabilization (MOMP). Bcl-2 family initiated MOMP causes release of the inter-membrane pro-apoptotic proteins to cytosol and creates a cytosolic environment suitable for the executionary phase of apoptosis. We designed the mathematical model of this regulatory network where the synthesis rates of the Bcl-2 family members served as the independent inputs. Using computational simulations, we have then analyzed the response of the model to up-/downregulation of the Bcl-2 proteins. Under several assumptions, and using estimated reaction parameters, a non-linear stimulus-response emerged, whose characteristics are associated with bistability and switch-like behavior. Interestingly, using the principal component analysis (PCA) we have shown that the given model of the Bcl-2 family interactions classifies the random combinations of inputs into two distinct classes, and responds to these by one of the two qualitatively distinct outputs. As we showed, the emergence of this behavior requires specific organization of the interactions between particular Bcl-2 proteins.

## Introduction

Programmed cell death (PCD), often denoted as a cellular suicide, plays an important role in the homeostasis of every multi-cellular organism [1]. One of the main forms of PCD is called apoptosis [1]–[3], a process which is well distinguished by its characteristic morphology [4]. Defects in apoptosis regulation may cause a variety of serious diseases, including the neurodegenerative disorders [5], autoimmune diseases [6], or cancer [7]–[9]. Apoptosis can be initiated by either extracellular stimuli or by signals originating from a cell's internal space [9], [10]. Signals initiating apoptosis then proceed through the apoptotic signaling pathways, which contain several control points [9], [10]. One of the most important of such points, integrating a multitude of incoming apoptotic (and antiapoptotic) signals is formed by a family of Bcl-2 (B-cell lymphoma 2) proteins [11], [12]. The Bcl-2 family controls mitochondrial outer membrane permeabilization (MOMP) [13], [14], the crucial event of apoptosis.

MOMP allows the release of key apoptotic players - Smac/DIABLO and cytochrome c, from a mitochondrial intermembrane space to cytosol [13], [14]. In the presence of ATP, released cytochrome c binds to a cytosolic protein Apaf-1, causing Apaf-1 oligomerization and recruitment of an inactive pro-caspase-9, leading to formation of a multi-protein complex known as an apoptosome [15]–[17]. Within the apoptosome, pro-caspase-9 subsequently undergoes processing and activation [15]–[17]. The active caspase-9 proteolytically activates caspase-3 [18]. Smac/DIABLO, once released to the cytosol, inhibits XIAP (X-linked inhibitor of apoptosis) - the most prominent suppressor of caspases-3 and -9 [19]. Caspase-3 and other effector caspases (caspases-6 and -7) are the primary executioners of the apoptosis [9], [20]. Activation of effector caspases signifies the point of no-return, after which apoptosis irreversibly occurs [21].

Bcl-2 family members are functionally classified as either antiapoptotic, or proapoptotic. Structurally, Bcl-2 proteins can be categorized according to the number of Bcl-2 homology domains (BH) in their *α*-helical regions [9], [22]. Antiapoptotic members (Mcl-1, A1, Bcl-xL, Bcl-2, Bcl-w and Bcl-B) are characterized by the presence of four BH domains (BH1-4) [23], [24]. Their role is to prevent MOMP by inhibition of proapoptotic family members [23], [24]. Proapoptotic members can be divided to BH3-only proteins and multidomain proteins - effectors [9]. BH3-only proteins can be further subdivided based on their role in apoptotic signaling. BH3-only subgroup members, termed sensitizers, or enablers (Noxa, Bad, Puma, Hrk, Bmf and Bik) can only bind to antiapoptotic Bcl-2 proteins, forming inactive dimers [22]. Members of another BH3 subgroup, termed activators (Bim and Bid), can act in the same way [22], but in addition, activators can directly activate effectors [23], [25]. Effectors, once activated, undergo oligomerization and form pores – mitochondrial apoptosis channels (MAC) in the mitochondrial outer membrane (MOM), leading eventually to MOMP. [14], [26]. Therefore, effectors are the primary target of inhibition by their antiapoptotic relatives [25].

Individual Bcl-2 family members are regulated by wide-variety of factors, e.g. growth factor deprivation, cytokine withdrawal, heat shock, DNA damage, hypoxia, death receptors stimulation and many others [23]. Mechanisms of regulation include transcription control and/or post-translational modifications by phosporylation, or proteolytic cleavage [23]. Bcl-2 family thus integrates a multitude of converging signals to decide whether to commit MOMP or not. This decision is carried in an all or nothing manner, giving no possibility of intermediate MOMP. This interesting behavior has made the Bcl-2 family an attractive subject of mathematical modeling and computer simulations.

There are several works regarding modeling and simulation of the Bcl-2 family and the control of MOMP, revealing and examining a variety of non-linear system behaviors such as robustness, stimulus-response ultrasensitivity [27] and steady-state bistability [28]–[30]. Besides these, the Bcl-2 family was involved in several other, more general models of apoptosis signaling [31]–[33].

In the above mentioned works, the authors reduce the complexity of their model by grouping of several functionally similar species together. Usually, the Bcl-2 family's members are assigned into four groups according to their structural and functional classification. The most prominent group's member is taken as the model's representation of the whole group of species. Previous models of the Bcl-2 regulatory network differ by level of details, nevertheless they all adopt such simplification. Although, such simplification provides an attractive trade-off between the model's complexity and plausibility, functional specificities of Bcl-2 family's individuals are being omitted.

In the proposed work we provide a literature-based mathematical model in which interactions between individual Bcl-2 family members are distinguished. Our goal was to investigate the behavior of the detailed model, in particular to prove/disprove the switching properties obtained by models based on functional grouping. In the process we obtained additional insight into the decision mechanism of the Bcl-2 control of MOMP. The non-trivial pattern-recognition emerged as a consequence of functional specificities of Bcl-2 family individuals. In addition, an explicit model of pair interactions allowed us to probe the pro- and anti-apoptotic potency of individual members of the Bcl-2 family and to rank them according to their ability to promote or prevent the MOMP event.

## Results

### Bistability of the Bcl-2 family regulation of MOMP

Several previous works [28]–[30] have been focused on the analysis of bistable behavior of the Bcl-2 family mediated regulation of MOMP. Similarly, we performed a variation of the single stimulus parameter of the model to analyze its steady-state stimulus-response dependence. The production rate of the tBid (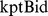) was considered as the stimulus and the steady-state concentration of the MAC (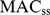) was the measure of response. The stimulus – 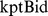 was varied through the chosen range of values, while the other parameters of the model remained unchanged.

**Figure 1:**
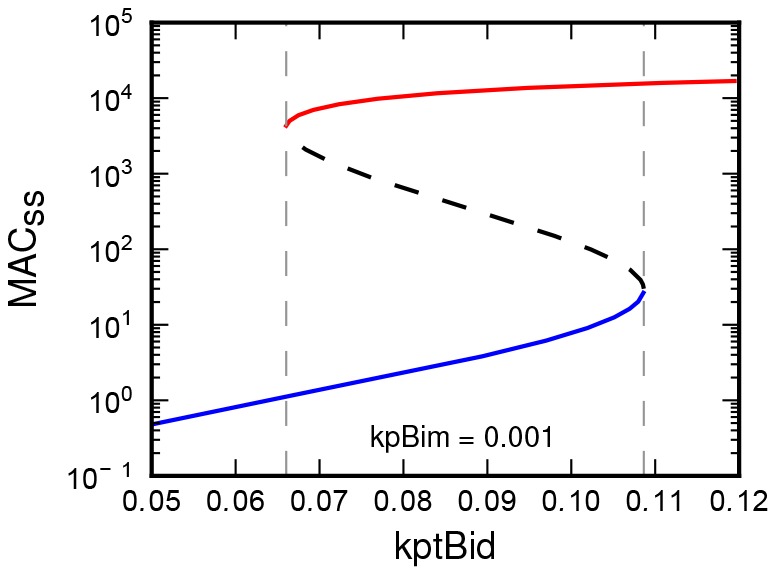
Steady-state concentration of the MAC (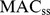) is plotted as a function of the production rate of tBid (kptBid). 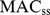
 is increasing with increasing value of 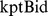. The 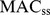 remains at very low levels (pro-survival – the blue solid curve), until the production rate exceeds the threshold (right vertical dashed line). Exceeding the threshold value causes sudden increase of the 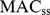 (onset of MOMP – red solid curve). The subsequent decrease of the production of tBid cause only slow decrease of 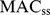, until the another threshold is crossed (left vertical dashed line). Then the 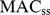 suddenly drops back to very low levels. Vertical dashed lines are enclosing the bistable region. Within this region system can persist in one of the two stable steady-states (solid curves), which are separated by unstable steady-states (dashed curve).

Using the default parameter setup of the model (see section Model and its biological relevance) we have obtained the stimulus-response dependence depicted in the Figure 1. The obtained steady-state stimulus-response forms hysteresis, the typical hallmark of bistability in dynamic system. The two thresholds (Fig. 1), marked by the left and right vertical dashed lines) enclose the bistable region. Within the bistable region, the system under the same value of the stimulus may occur in one of two stable steady-states, depending on the initial conditions. These steady-states are separated by the unstable steady-states (marked by dashed curve). Such systems are often described as the mechanism of a “toggle switch” [34].

Previous works limited themselves to the response of the Bcl-2 family induced by activators tBid/Bim solely. In the presented work we have explored the model's response to variation of all components, including both activators, all the anti-apoptotic proteins, enablers and effectors. Utilizing individual production rates as the input stimuli, we performed set of analyses analogous to the previous one.

**Figure 2:**
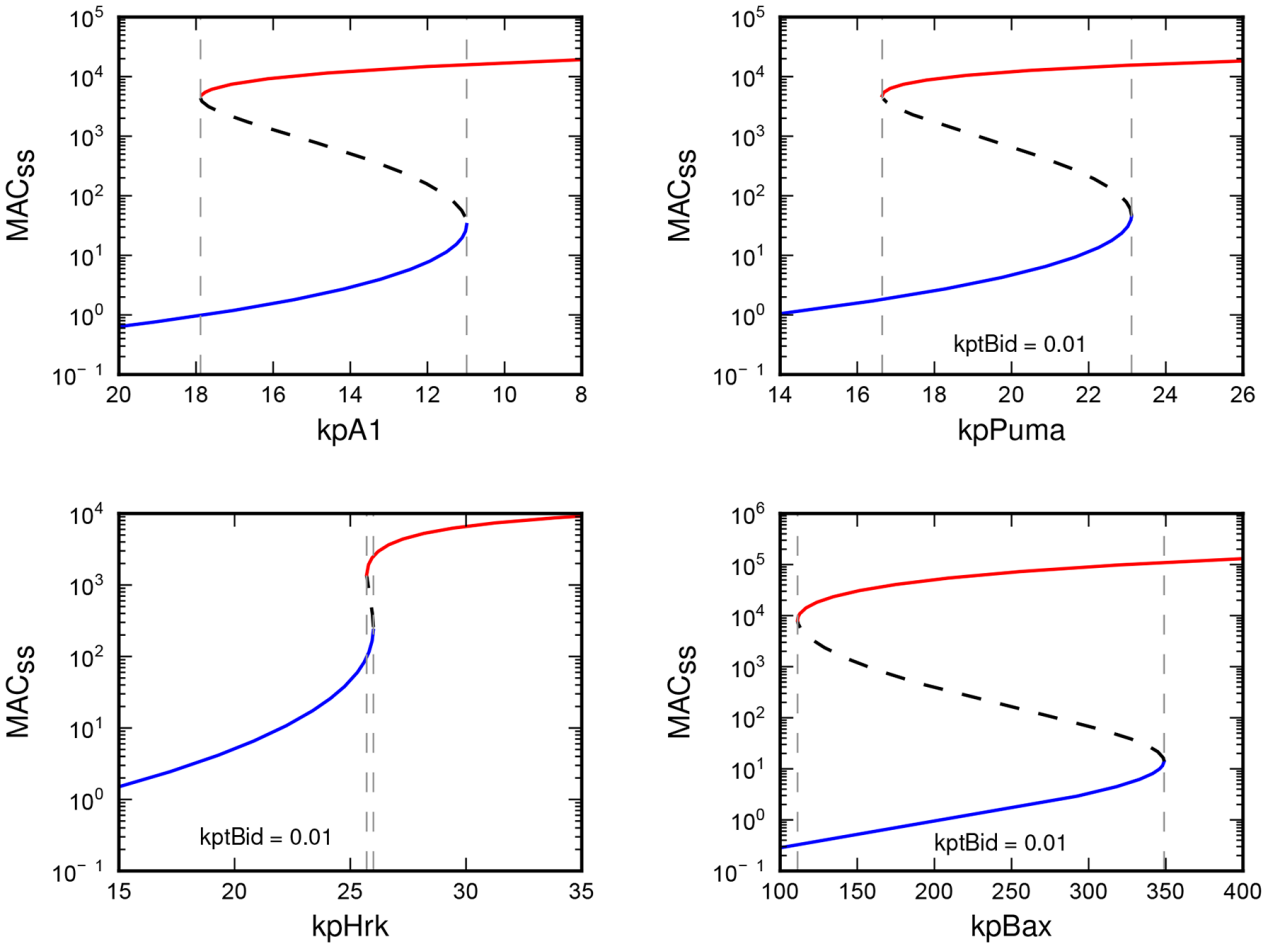
Steady-state concentration of the MAC plotted as a function of indicated production rates.

In addition to tBid, we have observed that steady-state stimulus-response hysteresis resulted also from stimulation by the second of activators – Bim (data not shown), as well as by the the anti-apoptotic proteins. Surprisingly, even a variation of the production rates of enablers Puma and Bad & Bmf yields stimulus-response hysteresis (see Fig. 2).

Variation of the Hrk & Bik productions yields hysteresis as well, but the obtained range of bistability is extremely narrow, close to ultrasensitive sigmoid curve. Similarly to bistability, a sigmoid curve indicates that the modeled system is insensitive to low levels of the given input, but that it can respond significantly high levels of the given input. In contrast to bistability, sigmoidal response is not discretized, as the response is continuously increasing/decreasing with the growing/reducing input strength. While the bistability can be compared to mechanism of “toggle switch” sigmoid stimulus-response is often compared to the functioning of the “push-button” [34].

Robust hysteresis with a wide bistable region was yielded by the variation of production rate of Bax and less pronounced hysteresis was obtained by variation of production of Bak. This shows that “toggle” switching of the Bcl-2 response can also be achieved by significant upregulation of the effector proteins.

**Figure 3:**
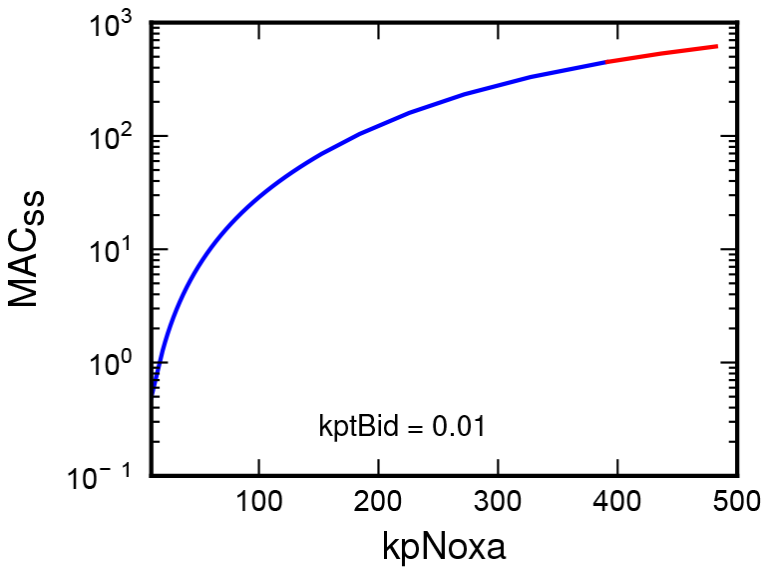
Steady-state concentration of the MAC is plotted as a function of the production rate of Noxa. Hyperbolic curve indicates a response sensitivity to even a small levels of stimulation.

Interestingly, variation of the production rate of the Noxa produced a hyperbolic response. Hyperbolic dependence, in contrast to hysteresis and sigmoid response, indicates sensitivity to even a small increase of input stimuli. Stimulation of the model through the changing production of Noxa, can therefore be viewed as “tuning” of the model's sensitivity to other incoming stimuli (see Fig. 3).

### Monte-Carlo variation of production rates results in bimodal distribution of steady-state abundance of MAC

In the previous section, we analyzed the dependence of the steady-state concentration of MAC on the production of individual proteins. In what follows, we performed Monte-Carlo analysis of the dependence of the steady-state concentration of MAC on the simultaneous variation of multiple production rates.

In each single iteration, the values of all the production rates (kpMcl1, kpA1, kpBclXl, kpBcl2, kpBclw, kpBclB, kpHrk, kpBik, kptBid, kpPuma, kpBim, kpBad, kpBmf, kpNoxa, kpBax, kpBak) of the model were simultaneously varied according to following rule:
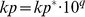
(1)where *kp* is the variated production rate, 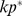 is its default value. and *q* is the uniformly distributed real number, randomly chosen at each iteration, and for each production rate, from interval 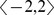. Other parameters of the model were kept at their default values. Similar to the analysis of bistability, the steady-state concentration of MAC, was used as the output.

**Figure 4:**
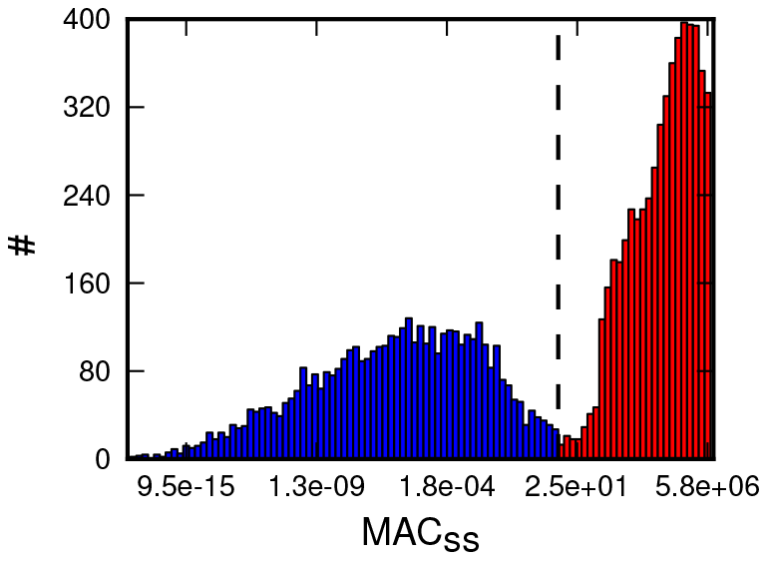
Distribution of steady-state concentrations of MAC produced by 10^4^ random variations of the model's production rates. Vertical dashed line denotes the minimum between two local maxima, defining the threshold value of the steady-state concentration of the MAC, distinguishing pro-survival and pro-MOMP responses.

We have plotted the distribution of the model's response obtained for 10^4^ iterations. The results (see Fig. 4) show clear bimodal distribution of the model's response. The bimodal distribution of the response proves that the model of Bcl-2 regulatory network can turn random combinations of inputs into two qualitatively distinct outputs [29], [35]–[37].

The minimum located between the local maxima of the response distribution (Fig. 4, marked by vertical dashed line) was considered as the threshold value of steady-state concentration of MAC, separating the pro-survival (colored blue) and MOMP (colored red) responses. Hereafter, the steady-state concentrations of the MAC below this threshold are considered as pro–survival, while the concentrations above the threshold are considered as MOMP initiating (pro–MOMP). It is worth mentioning, that the value of the threshold intersects all the hysteresis curves (Fig. 1 and 2), within the bistable range. The value of this threshold – ∼350, assumes that MOMP occurs once the number of effector dimers in mitochondria surrounding environment exceeds 350. This is remarkably good agreement with experimental estimations made by Martinez-Caballero et al. [38].

### Bcl–2 family performs non-trivial pattern recognition

Using the same sets of the production rates as were used in the previous analysis we arranged the matrix of stimuli. Each row of this matrix – stimuli vector corresponds to one iteration of the Monte-Carlo analysis from the previous section and each of its columns corresponds to the one of the production rates, defining the size of the stimuli matrix to 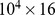. Each column was normalized to its mean. Then we performed the principal component analysis (PCA) of the matrix of stimuli and plotted the input vectors within the plane defined by principal components.

**Figure 5:**
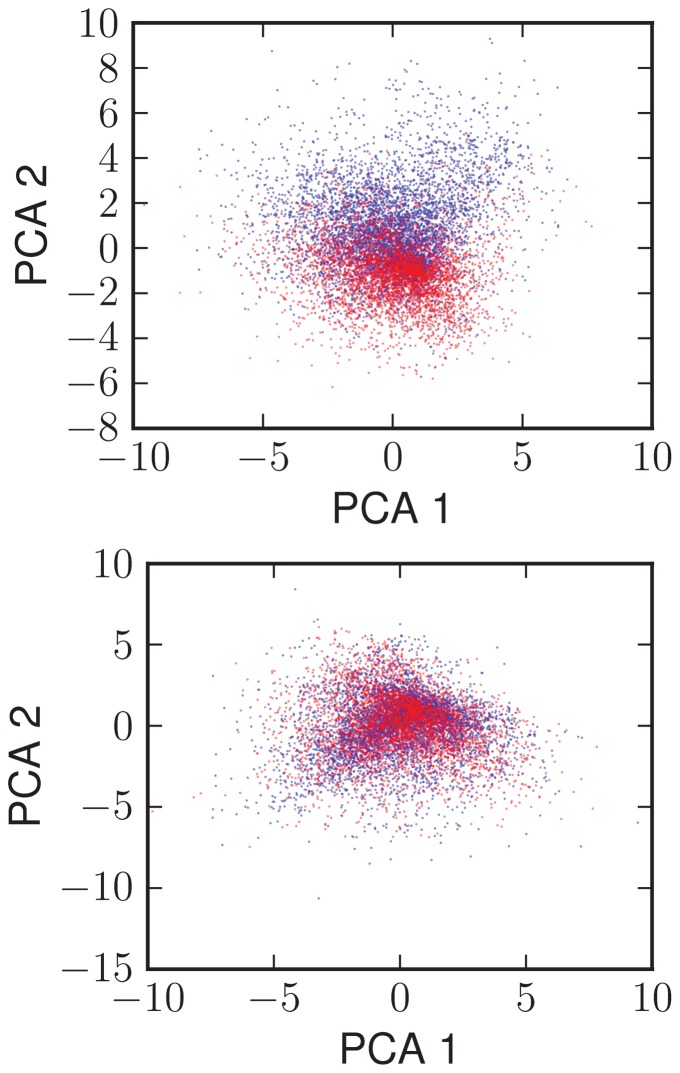
Scatter plot of all input combinations, plotted in the plane defined by the principal components analysis. Inputs associated with pro-MOMP response are colored red, remaining inputs are colored blue. The clusterization of inputs according to response quality is apparent for reference model (top), but obviously absent when altering the model's topology (bottom).

The results (see Fig. 5, top) show that when the random input stimuli vectors are plotted in the PCA–defined plane, they form a randomly scattered cloud. But, when the each vector is colored based on the associated response, it appears that the stimuli associated with the given response are clustered. This shows, that the model of Bcl-2 regulatory network is capable of taking a wide range of random combinations of incoming signals and classifying them into two sharply defined responses of distinct biological relevance. Such functionality defines what is in the field of machine learning and neural networks known as non-trivial pattern recognition [39]–[42].

In following, we created ten alternative models of the Bcl-2 family, by mutating the topology of the default model. By mutation we mean random addition of non-existing or deletion of existing inhibitory interactions between anti-apoptotic and pro-apoptotic proteins and/or activations of effectors by activators. Such mutations allow alteration of the Bcl-2 family model on its detailed level, but preserve the interactions between the functional groups consistent with the default model.

For each of the alternative models we have performed a total of five of such mutations. We then performed Monte-Carlo analysis of these models by generating the 10^4^ random combinations of input stimuli. For each alternative model we then identified the threshold value of the effectors activity and subsequently performed the PCA of the stimuli matrix.

As a result we have found that all the alternative models produced bimodal distribution of response, but none of them clustered input stimuli similarly to the default model (see Fig. 5, bottom). This indicate that, while the bistability can emerge from alternative topologies of the Bcl-2 family interaction network, the pattern recognition is strictly associated with this particular topology of this regulatory network.

### Pivotal pro- and anti-apoptotic Bcl-2 family members

In the following we wanted to answer the naturally arising question: Which of the production rates are pivotal regarding the determination of the model's response?

The model's response varies over several orders of magnitude. However, all the response values below/above the threshold, regardless of the value itself, are considered to be qualitatively equal – pro–survival/pro–MOMP, providing the same biological consequences. Therefore, we were interested in correlation of the values of the production rates with the response quality (pro–survival/pro–MOMP), instead of the correlation with the response quantity.

We utilized the point-biserial correlation coefficient (PBCC) - 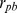 as the the measure of the correlation between the value of the production rate and quality of the model's response (pro–survival/pro–MOMP). PBCC is frequently used to measure the correlation between the two variables, one of which is dichotomous (either naturally, or artificially dichotomized) [43]. PBCC for each production rate was calculated as follows:
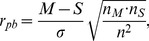
(2)where the *M* and *S* are the means of values of the given production rate corresponding to pro–MOMP and pro–survival responses respectively. 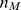 and 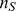 are the number of values of the given production rate, corresponding to pro–MOMP and pro-survival responses respectively. *n* is the total number of the values of the given production rate and *σ* is its standard deviation.

**Figure 6:**
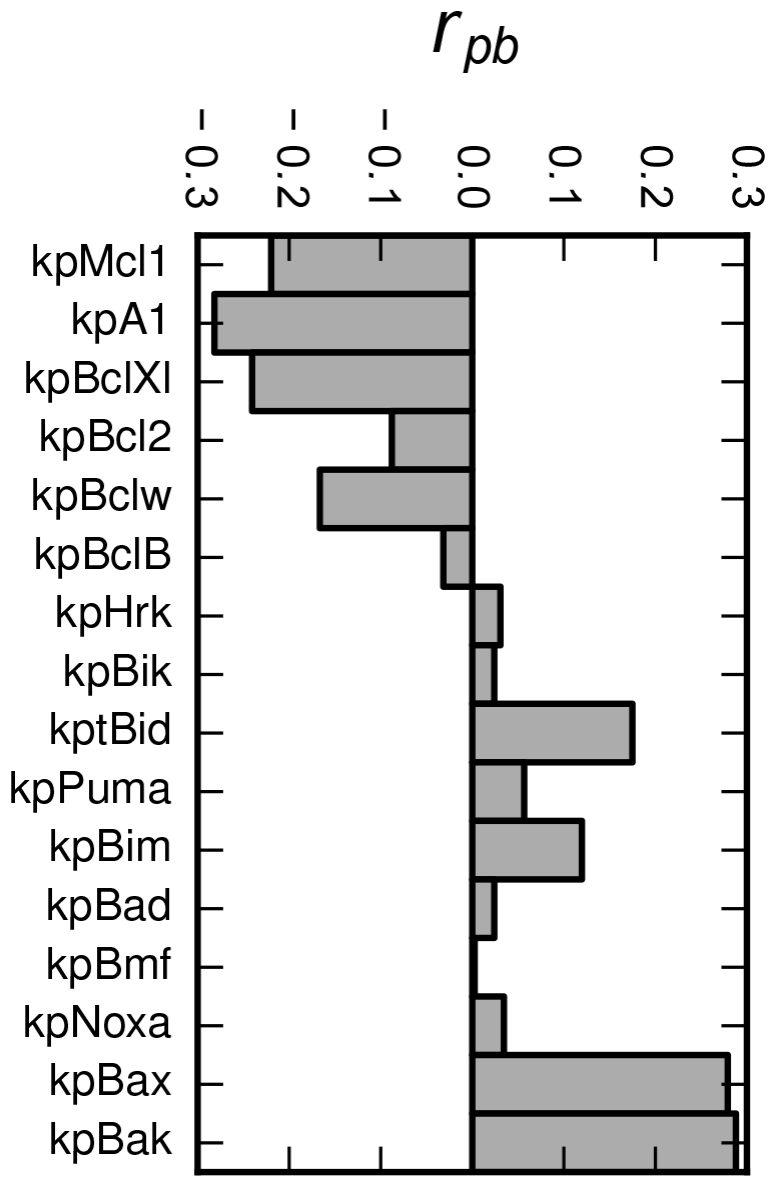
Point-biserial correlation coefficients (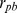) as the measure of correlation between the values of the production rates and the model's response.

The results we obtained (Fig. 6) prove the role of the tBid and Bim – the only activators of effectors – as the primary pro-apoptotic proteins. Similarly, the Puma is the most efficient MOMP promoter among the enabler proteins within the model. On the other hand, as model predicts, the most efficient MOMP preventers are proteins A1 and Bcl-Xl, followed by Mcl-1 and Bcl-w.

## Discussion

The Bcl-2 family of proteins consists of sixteen (excluding putative tissue-specific Bcl-2 protein known as Bcl-2 ovarian killer – Bok) proteins that differ in their upstream regulation as well as in their interactions with other family members. We translated current biological knowledge of these interactions into mathematical model, which was utilized to study the regulation of the MOMP – crucial apoptotic event, which is maintained by the Bcl-2 family. Under given assumptions, that have been made to construct the model (see Sec. Model and its biological relevance), and using estimated parameters, interesting behaviors have been found to emerge from Bcl-2 protein interactions.

Analysis of the model's response to variation of productions of individual proteins, revealed that the system property called steady-state bistability emerges as a robust feature of the modeled system. Our model predicts that the most of the Bcl-2 proteins can potentially serve similarly to bistable “toggles”, up- or downregulation of these cause “switching on” the MOMP. Steady-state bistability is currently being, the favorite framework for thinking about the switch between life and death” [44]. Therefore, the above-mentioned results were expected. However, we have also found that other Bcl-2 proteins can potentially act as a “push-buttons” – alternative switching mechanisms with very narrow or missing bistable range and one of them as a “tuner” of the model's sensitivity – upregulation of which results in hyperbolic model's response.

We have shown that the model is able to process random combinations of inputs to produce output that has bimodal distribution. This strongly suggests that orchestration of these “toggles”, “push-buttons” and “tuners” can constitute a molecular device whose function is to integrate multitude of incoming, continuous inputs into binary outcomes. Bimodal distribution was previously observed by Sun et al [29] in the flow cytometry of Bax activation as the response to staurosporine treatment of the HeLa cells population, supporting our finding.

Moreover, our model of this molecular device shows ability to perform pattern recognition – which is non-trivial functionality, often associated with neural networks and machine learning algorithms. This functionality is impaired by deletion of a relatively small number of reactions, as well as by addition of artificial interactions, even for interactions which are consistent with the relationships between the functional groups of the family.

Measurements of the correlation between the individual signals and the model's response predict that the most potent inputs of this network are associated with the regulation of activators tBid and Bim, enabler protein Puma and the anti-apoptotic sentinels A1, Bcl-Xl, Bcl-w and Mcl-1.

Finally, to outline the directions in which the proposed work could be extended, we would like to point out the necessity of utilization of more sophisticated, machine-learning based approaches to better analyze the synergy of the Bcl-2 family regulation of MOMP. We believe that its understanding can be crucial in development of novel anti-cancer drugs and/or treatment of the serious neurodegenerative diseases.

### Model and its biological relevance

In the proposed work, we modeled the MOMP regulatory network formed by the interactions of the members of the Bcl-2 family of proteins (see Table 1). Our model of the Bcl-2 family is defined by the extensive set of reactions which are listed in Table 2 and the respective set of reaction rates (Table 3). In following we describe important features of our model, giving special emphasize to the assumptions and simplification which we have made toward the model feasibility and simplicity.

**Table 1:** Interactions between individual members of the Bcl-2 family of proteins.

Group & Member	Binds to & inhibits:	Activates:	Ref.
*Antiapoptotic*			
*proteins:*			
Mcl-1	Noxa, Bim, Puma, Bax, Bak		[9], [47]
Bcl-2	Bad, Bim, Puma, Bmf, Bax		[9], [47]
A1	Noxa, Bim, Puma, Bid, Hrk, Bik, Bax, Bak		[9], [47]
Bcl-xL	Bad, Bim, Puma, Bid, Hrk, Bmf, Bik, Bak, Bax		[9], [47]
Bcl-w	Bad, Bim, Puma, Bid, Hrk, Bmf, Bik, Bax		[9], [47]
Bcl-B	Bax		[48]
*Enablers:*			
Noxa	Mcl-1, A1		[9], [22], [47]
Bad	Bcl-xL, Bcl-w, Bcl-2		[9], [22], [47]
Puma	Bcl-xL, Bcl-w, Bcl-2, Mcl-1, A1		[9], [22], [47]
Hrk	Bcl-xL, Bcl-w, A1		[47]
Bmf	Bcl-xL, Bcl-w, Bcl-2		[22], [47]
Bik	Bcl-xL, Bcl-w, A1		[47]
*Activators:*			
Bim	Bcl-xL, Bcl-w, Bcl-2, Mcl-1, A1	Bax, Bak	[9], [22], [47]
tBid	Bcl-xL, Bcl-w, A1	Bax, Bak	[22], [47]
*Effectors:*			
Bak	Bcl-xL, Mcl-1, A1		[9], [22]
Bax	Bcl-xL, Bcl-w, Bcl-2, Bcl-B, Mcl-1, A1		[9], [22]

**Table 2:** List of reactions of the model of the Bcl–2 family of proteins.

No.	Reaction	Forward rate	Reverse rate
1	tBid+Bax 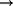 tBid+aBax	ka	
2	tBid+Bak 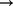 tBid+aBak	ka	
3	Bim+Bax 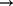 Bim+aBax	ka	
4	Bim+Bak 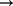 Bim+aBak	ka	
5	Mcl1+Puma 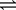 Mcl1∼Puma	ks	km
6	Mcl1+Bim 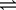 Mcl1∼Bim	ks	km
7	Mcl1+Noxa 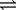 Mcl1∼Noxa	ki	km
8	A1+Hrk 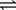 A1∼Hrk	ki	km
9	A1+Bik 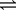 A1∼Bik	ki	km
10	A1+tBid 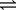 A1∼tBid	ks	km
11	A1+Puma 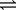 A1∼Puma	ks	km
12	A1+Bim 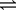 A1∼Bim	ks	km
13	A1+Noxa 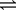 A1∼Noxa	kw	km
14	BclXl+Hrk 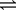 BclXl∼Hrk	ks	km
15	BclXl+Bik 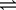 BclXl∼Bik	ki	km
16	BclXl+tBid 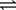 BclXl∼tBid	ki	km
17	BclXl+Puma 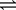 BclXl∼Puma	ks	km
18	BclXl+Bim 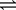 BclXl∼Bim	ks	km
19	BclXl+Bad 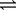 BclXl∼Bad	ks	km
20	BclXl+Bmf 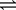 BclXl∼Bmf	ks	km
21	Bcl2+Hrk 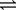 Bcl2∼Hrk	kw	km
22	Bcl2+Bik 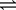 Bcl2∼Bik	kw	km
23	Bcl2+Puma 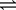 Bcl2∼Puma	ks	km
24	Bcl2+Bim 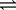 Bcl2∼Bim	ks	km
25	Bcl2+Bad 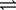 Bcl2∼Bad	ki	km
26	Bcl2+Bmf 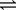 Bcl2∼Bmf	ks	km
27	Bclw+Hrk 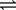 Bclw∼Hrk	ki	km
28	Bclw+Bik 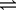 Bclw∼Bik	ki	km
29	Bclw+tBid 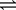 Bclw∼tBid	ki	km
30	Bclw+Puma 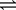 Bclw∼Puma	ks	km
31	Bclw+Bim 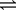 Bclw∼Bim	ks	km
32	Bclw+Bad 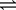 Bclw∼Bad	ki	km
33	Bclw+Bmf 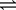 Bclw∼Bmf	ks	km
34	Mcl1+aBax 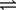 Mcl1∼aBax	ks	km
35	Mcl1+aBak 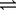 Mcl1∼aBak	ks	km
36	A1+aBax 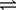 A1∼aBax	ks	km
37	A1+aBak 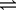 A1∼aBak	ks	km
38	BclXl+aBax 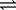 BclXl∼aBax	ks	km
39	BclXl+aBak 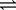 BclXl∼aBak	ks	km
40	Bcl2+aBax 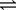 Bcl2∼aBax	ks	km
41	Bclw+aBax 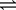 Bclw∼aBax	ks	km
42	BclB+aBax 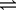 BclB∼aBax	ks	km
43	aBax+aBax 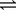 aBax∼aBax	kd	km
44	aBak+aBak 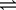 aBak∼aBak	kd	km
45	aBax+aBak 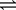 aBax∼aBak	kd	km
46	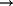 Hrk	kpHrk	
47	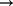 Bik	kpBik	
48	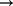 tBid	kptBid	
49	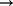 Puma	kpPuma	
50	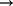 Bim	kpBim	
51	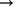 Bad	kpBad	
52	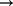 Bmf	kpBmf	
53	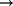 Noxa	kpNox	
54	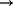 Mcl1	kpMcl1	
55	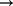 A1	kpA1	
56	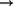 BclXl	kpBclXl	
57	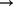 Bcl2	kpBcl2	
58	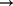 Bclw	kpBclw	
59	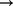 BclB	kpBclB	
60	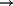 Bax	kpBax	
61	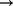 Bak	kpBak	
62	(All) 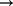	kdeg	

Reactions no. 46–61 denote production of the correspondent species, while the reaction no. 62 denotes degradation of all the species of the model.

**Table 3:** List of parameters of the model of the Bcl–2 family of proteins.

Param.	Default value		Notes & Ref.
	[ 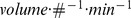 ]	[ 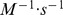 ]	
ks	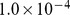	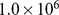	[47]
ki	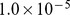	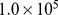	[47]
kw	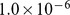	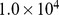	[47]
ka	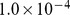	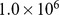	estimated
kd	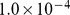	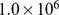	estimated
	[ 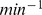 ]	[ 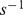 ]	
km	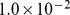	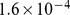	estimated
kdeg	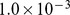	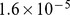	estimated
	[ 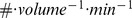 ]	[ 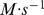 ]	
kpHrk	1.0	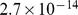	1/6 of 6.0, [28], [50], [51]
kpBik	1.0	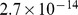	1/6 of 6.0, [28], [50], [51]
kptBid	0.1	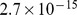	1/2 of 0.2, [28], [50], [51]
kpPuma	1.0	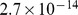	1/6 of 6.0, [28], [50], [51]
kpBim	0.1	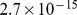	1/2 of 0.2, [28], [50], [51]
kpBad	1.0	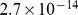	1/6 of 6.0, [28], [50], [51]
kpBmf	1.0	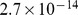	1/6 of 6.0, [28], [50], [51]
kpNoxa	1.0	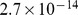	1/6 of 6.0, [28], [50], [51]
kpMcl1	10.0	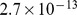	1/6 of 60.0, [28], [52], [53]
kpA1	10.0	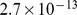	1/6 of 60.0, [28], [52], [53]
kpBclXl	10.0	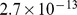	1/6 of 60.0, [28], [52], [53]
kpBcl2	10.0	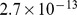	1/6 of 60.0, [28], [52], [53]
kpBclw	10.0	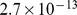	1/6 of 60.0, [28], [52], [53]
kpBclB	10.0	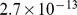	1/6 of 60.0, [28], [52], [53]
kpBax	60.0	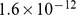	1/2 of 120.0, [28], [52], [53]
kpBak	60.0	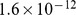	1/2 of 120.0, [28], [52], [53]

Default values of parameters are listed using model-specific units (left column) as appear in the PySCes model definition file and using common units (right column). Values were recalculated assuming 1 nM = 600 molecules per abstract reaction volume (similarly to work of Eissing et al. [49]). Units differ between parameters as these corresponds to reactions of different order. Default values of the production rates were calculated as respective fractions of production rates which were estimated from references and which apply to respective Bcl-2 protein classes.

In each simulation, the initial abundance of each protein was either set to zero for a protein that is not synthesized from external sources (reactions 46–61), otherwise defined by following rule:
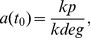
(3)setting the initial abundance to balance the production (reactions 46–61) and degradation of the given protein at the initial conditions. Bcl-2 proteins' productions, as appear in our model, involve *de-novo* synthesis of the particular protein and may also involve the post-translational activation of this protein by external cellular signals (as is the case of the Bid which is truncated by caspase-8 to active tBid). Although synthesis of the Bcl-2 proteins could potentially by regulated by activity of other Bcl-2 family relatives on the post-transcriptional level, we are not aware whether any mechanistic details of such kind of intra-familiar regulation are currently known, and therefore we assume the production rates to be fully independent of the Bcl-2 proteins activity. Similar assumption is often made in similar models, as was also made in the previous models of the Bcl-2 apoptotic switch (e.g. Cui et al. [28]). Protein productions can thus serve as an independent, multivariate input, model's response to which we study by computational simulations.

Degradation half-life times of the Bcl-2 proteins were reported to range between 15–45 minutes (Mcl-1) and from 12 to more than 24 hours (Bax) [45]. However, to reduce number of parameters and reduce the complexity of the model, degradation rate of all proteins was modeled by catch-all reaction (catch-all reaction 62) and set to resemble the ∼10 hours degradation half-life time. Although this simplification was found to affect the obtained results only quantitatively (data no shown), it needs to be highlighted as a difference from biological reality.

The abundance of the mitochondrial apoptosis channels – MACs of the model was calculated as the sum of the abundances of the aBax∼aBax, aBak∼aBak, aBax∼aBak dimers. This is an important simplification, as MACs are reported to comprise usually several to several tens of monomeric units [38]. Similar simplification is, however, often adopted by modeling works, and was also used in several previous models of the Bcl-2 apoptotic switch (e.g. Cui et al. [28] and Sun et al. [29]).

Default values of the reaction rates (listed in the Table 3) were estimated according to typical rates of biomolecular interactions of similar type, and in accord with the previously published models of the Bcl-2 family [27]–[30]. It has to be noted that there has been no analysis of the model's parametric robustness performed yet, and thus the obtained results must be interpreted as valid only under the given parametrization and on the basis of given assumptions.

Protein interactions were modeled using mass action kinetics, translated into a set of ordinary differential equations. The model was implemented and all the analyses were performed within Python programming language, by using the PySCes (Python Simulator for Cellular Systems) [46] module.
